# The Importance of Vibronic Coupling for Efficient Reverse Intersystem Crossing in Thermally Activated Delayed Fluorescence Molecules

**DOI:** 10.1002/cphc.201600662

**Published:** 2016-07-26

**Authors:** Jamie Gibson, Andrew P. Monkman, Thomas J. Penfold

**Affiliations:** ^1^ School of ChemistryNewcastle UniversityNewcastle upon TyneNE1 7RUUK; ^2^ School of PhysicsUniversity of DurhamDurhamDH1 3LEUK

**Keywords:** light-emitting diodes, reverse intersystem crossing, thermally activated delay fluorescence, vibronic coupling, quantum dynamics

## Abstract

Factors influencing the rate of reverse intersystem crossing (*k*_rISC_) in thermally activated delayed fluorescence (TADF) emitters are critical for improving the efficiency and performance of third‐generation heavy‐metal‐free organic light‐emitting diodes (OLEDs). However, present understanding of the TADF mechanism does not extend far beyond a thermal equilibrium between the lowest singlet and triplet states and consequently research has focused almost exclusively on the energy gap between these two states. Herein, we use a model spin‐vibronic Hamiltonian to reveal the crucial role of non‐Born‐Oppenheimer effects in determining *k*_rISC_. We demonstrate that vibronic (nonadiabatic) coupling between the lowest local excitation triplet (^3^LE) and lowest charge transfer triplet (^3^CT) opens the possibility for significant second‐order coupling effects and increases *k*_rISC_ by about four orders of magnitude. Crucially, these simulations reveal the dynamical mechanism for highly efficient TADF and opens design routes that go beyond the Born‐Oppenheimer approximation for the future development of high‐performing systems.

The communication between low‐lying singlet and triplet excited states is of great importance in the field of organic electronics and plays a fundamental role in determining key molecular and material properties such as the lifetime and mobility of charge‐separated states.^1^, ^2^ Consequently, a thorough understanding of the basic principles governing the interplay between these manifolds of spin states is of great importance, with direct implications for the performance of devices such as organic photovoltaics and organic light‐emitting diodes (OLEDs).

For the latter this has particular significance owing to the emergence of thermally activated delayed fluorescence (TADF).^3^ In OLEDs, electrical excitation of the emitting molecules generates, through spin statistics, a 3:1 ratio of triplet and singlet excited states. Therefore, efficient devices call for an effective mechanism for harvesting the triplet states, which in fluorescence molecules are simply lost to non‐radiative processes. Presently, the most popular approach is to exploit the large spin orbit coupling constant of iridium complexes to harvest the triplet population by phosphorescence.^4^ However, as iridium is the fourth most scarce naturally occurring element on earth, it is unsustainable to base large‐scale high‐volume industries, such as lighting, on this resource.

Adachi and co‐workers^7^, ^8^ recently demonstrated the effectiveness of the TADF approach for third‐generation heavy‐metal‐free OLEDs. This harvests the triplet excited states by exploiting thermal energy to drive population transfer from the triplet to the singlet states so that they can emit as singlet states via delayed fluorescence. These systems can therefore achieve a high luminescence quantum yield using only organic molecules. Adopting the predictions of Beens and Weller,^9^ Adachi and co‐workers demonstrated that TADF emitters could be constructed using charge‐transfer (CT) complexes, which exhibit a negligible gap between the singlet and triplet states due to the small exchange energy splitting.^10^, ^11^ This approach has subsequently been exploited^12^ to achieve efficient OLEDs with 100 % internal quantum efficiency and >30 % external quantum efficiency.^13^

Despite the intense research interest and obvious promise of TADF materials, it is astounding that the reverse intersystem crossing (rISC) process, crucial to TADF is not fully understood. This absence is important for achieving rational material design and especially given the widening scope of TADF in applications such as fluorescence imaging.^14^ One of the first reports of TADF was by Parker et al.^15^ The authors rationalised their findings in terms of a thermal equilibrium between the lowest singlet and triplet states [Eq. (1)]:

(1) $$K=\frac{\left[S_{1}\right]}{\left[T_{1}\right]}=\frac{k_{\mathrm{rISC}}}{k_{\mathrm{ISC}}}=\frac{1}{3}exp\left(-\Delta E_{S_{1}-T_{1}}/k_{b}T\right)$$

making it possible to calculate the rate of the whole TADF process (*k*_TADF_), that is, rISC proceeded by fluorescence as [Eq. (2)]:

(2) $$k_{\mathrm{TADF}}=\frac{1}{3}k_{f}exp\left(-\Delta E_{S_{1}-T_{1}}/k_{b}T\right)$$

This approach, adopted by Adachi and co‐workers, defines the TADF equilibrium as depending exclusively on the energy gap between the singlet and triplet states and crucially, as shown in Equation (1), independent of the coupling between them, that is, *k*_ISC_ and *k*_rISC_, provided it is not zero. This strongly motivates design procedures that focus upon minimising this energy gap using CT states and suppressing larger amplitude molecular vibrations to reduce non‐radiative pathways.^16^

However, the key assumption in this equilibrium description, that is, *k*_ISC_ and *k*_rISC_≫*k*_f_ must be broken to support new emitters with stronger fluorescence yields. Within this regime, TADF is cast in terms of a kinetic process,^17^ which in the long time limit, assuming non radiative and phosphorescence channels are small can be expressed as [Eqs. (3a) and (3)]:

(3) $$\frac{dS_{1}}{\mathrm{dt}}=-k_{f}\left[S_{1}\right]+k_{\mathrm{rISC}}\left[T1\right]$$

(4) $$\frac{dT_{1}}{\mathrm{dt}}=-k_{\mathrm{rISC}}\left[T1\right]$$

Solving Equations (3a) and (3b) yields Equations (4a) and (5):

(5) $$T_{1}\left(t\right)=T_{1}\left(0\right)exp\left(-k_{\mathrm{rISC}}t\right)$$

(6) $$S_{1}\left(t\right)=\left(\frac{k_{\mathrm{rISC}}}{k_{f}-k_{\mathrm{rISC}}}\right)T_{1}\left(0\right)exp\left(-k_{\mathrm{rISC}}t\right)$$

where *T*_1_(0) is the population of the triplet states at *t*=0, *S*_1_(*t*) and *T*_1_(*t*) are the time‐dependent populations of the emitting singlet and triplet states, respectively. This shows that *k*_rISC_ is crucial in determining the effectiveness of TADF. It is also important within a device context for the roll‐off efficiency of TADF OLEDs.^18^ Further supporting the breakdown of the thermal equilibrium representation, Ward et al.^19^ recently showed that different D–A–D molecules with very similar energy gaps (Δ*E* ${}_{S{}_{1}-T{}_{1}}$ ) exhibit large variations in *k*_rISC_. They found that by sterically hindering the motion of D and A group, the emission can be switched from TADF to phosphorescence. This effect cannot be explained within present models for TADF and is indicative of a mechanism which is dynamic in nature, in the sense that it depends on molecular vibrations.

Another aspect that remains unclear is the coupling responsible for the rISC. Spin orbit coupling (SOC) between ^1^CT and ^3^CT of D–A complexes is forbidden.^20^ It is therefore unlikely for the ^1^CT state to harvest the population of the ^3^CT states via rISC directly, especially in view of the large *k*_rISC_≈10^7^ s^−1^ reported.^21^, ^22^ Alternatively Ogiwara et al.^23^ used electron paramagnetic resonance (EPR) spectroscopy to propose that rISC is driven by hyperfine coupling induced ISC. This mechanism arises from interactions between an electron′s spin and the magnetic nuclei of its molecule. It is therefore completely local and not quenched by significant electron‐hole separation experienced in CT states like SOC.^24^ However, the hyperfine coupling constants are very small, usually in the range of 10^−4^ meV, and it therefore also appears highly unlikely that such coupling accounts for these recent observations. Recently, D–A and D–A–D molecular TADF systems^19^, ^21^, ^25^ have demonstrated that two of the excited states involved in the rISC step can be independently tuned. They must therefore be of different character and a ^1^CT and local excitonic triplet (^3^LE) pair appears most likely. Indeed, SOC between these two sets of states will be allowed.

Two theoretical papers have recently discussed the influence of multiple excited states and vibrational motion on TADF.^26^, ^27^ Firstly, Chen et al.^26^ used Fermi′s Golden Rule to calculate the rate of rISC occurring via SOC. They found that their calculated value differed significantly from experimental values, even for the ^1^CT→^3^LE transition. They subsequently postulated that for D–A–D systems this deviation occurs due to an absence of nonadiabatic effects between the low‐lying excited states involved in the upconversion. Marian^27^ then used multi‐reference quantum chemistry methods to show, in agreement with ref. 26 that direct SOC was indeed too small to explain efficient rISC. Instead Marian proposed that it is mediated by mixing with an energetically close‐lying ^3^LE state along a carbonyl stretching mode. Both of these works, which are largely consistent with previous experimental findings, promote mixing between multiple excited states as being crucial to efficient rISC. However, neither explicitly calculate the dynamical processes important to understand the exact mechanism of rISC. Consequently, to construct a mechanistic understanding of the TADF process, and crucially the *k*_rISC_, herein we use model quantum dynamics simulations to investigate the role of vibronic (nonadiabatic) coupling on *k*_ISC_ and *k*_rISC_. This is achieved using a D–A molecule composed of a phenothiazine donor and a dibenzothiophene‐S,S‐dioxide acceptor (PTZ‐DBTO2), shown in Figure 1.^5^ As also shown in ref. 5 this dimer analogue and the D–A–D trimer both give identical photophysics and excellent OLED performance >19 % EQE. Our Hamiltonian, composed of the most important electronic and vibrational degrees of freedom, enables us to demonstrate the crucial role of non Born‐Oppenheimer effects in determining these rates and the mechanism by which efficient rISC occurs. We show efficient rISC cannot be achieved using direct coupling between two states, but must incorporate a third.

**Figure 1:**
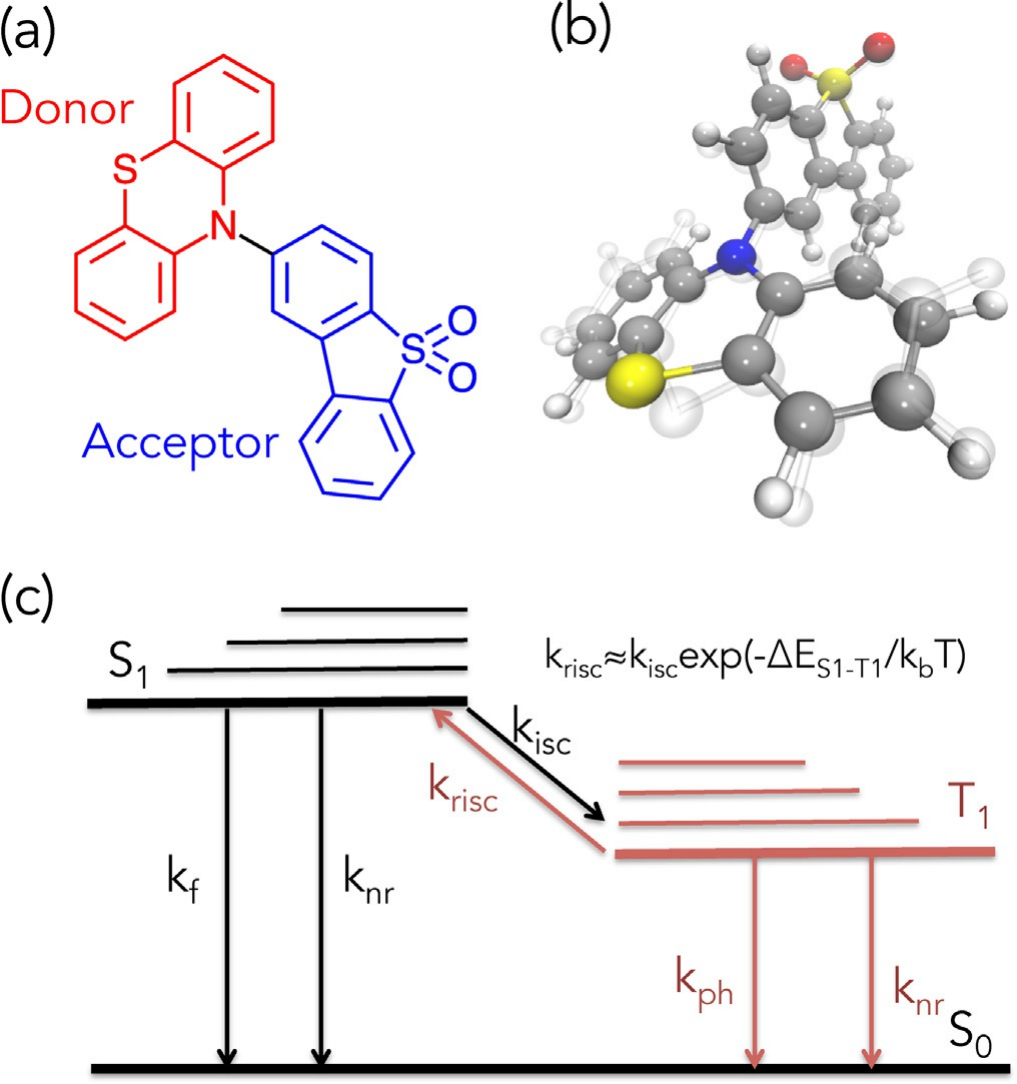
a) Schematic representation of the donor–acceptor (D–A) molecule composed of a phenothiazine donor and a dibenzothiophene‐S,S‐dioxide acceptor (PTZ‐DBTO2).^5^ b) A 3D structure of the ground state of PTZ‐DBTO2.^5^ The transparent overlaid structure is the optimised geometry in the S_1_ state. c) A simplified energy diagram representing a general schematic of the the up‐conversion of triplet states to a higher energy singlet state. *k*_rISC_ is approximately equal to the *k*_ISC_ multiplied by a Boltzmann factor determine the number of states with sufficient energy to overcome barrier (Δ*E* ${}_{S{}_{1}-T{}_{1}}$ ). Deviations from this relationship arise from a higher density of final states expected for the direct intersystem crossing case.^6^

The most relevant low‐lying valence excited states of PTZ‐DBTO2 are the lowest singlet CT state (^1^CT), the lowest triplet CT state (^3^CT) and the lowest triplet LE state (^3^LE). In agreement with recent experimental findings,^5^, ^19^ our TDDFT simulations show that the lowest triplet state is the ^3^LE on the donor group composed of a HOMO→LUMO+3 (Figure 2 a) transition. The two charge transfer states (^3^CT and ^1^CT) are dominated by a HOMO→LUMO transitions. The energies of these states, shown in Table S2 of the Supporting Information (SI), are in good agreement with the absorption spectra shown in ref. 5.

**Figure 2:**
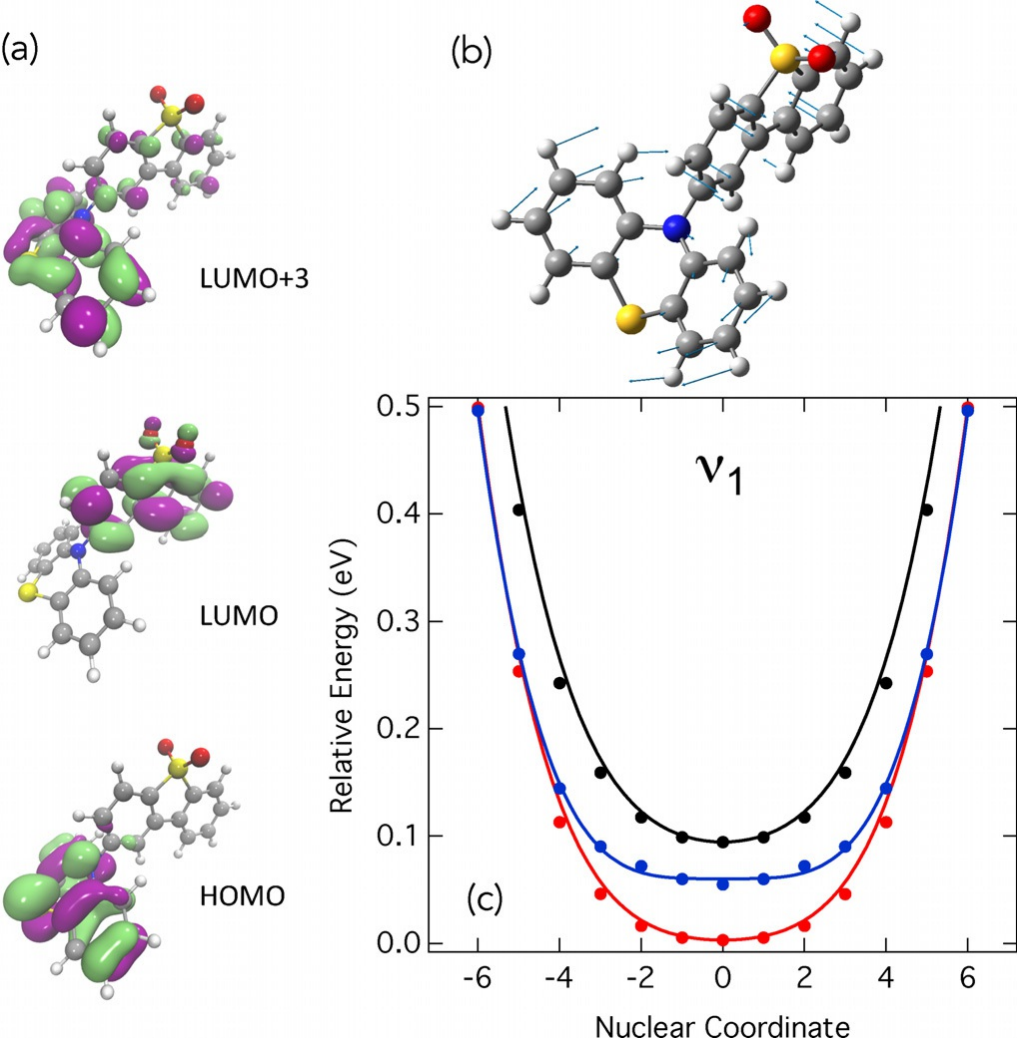
a) Molecular orbitals most involved in the low‐lying excited states (^3^LE, ^3^CT and ^1^CT) considered herein. b) Nuclear displacements, corresponding to a torsion of the D–A angle, associated with the lowest molecular normal mode (*v*_1_) of PTZ‐DBTO2. c) Potential energy curves of the low‐lying excited states (^3^LE=red, ^3^CT=blue and ^1^CT=black) relative to the ^3^LE energy minimum calculated using TDDFT (dots). The solid lines corresponds to the fit of the model vibronic coupling Hamiltonian to these potentials.

Figure 2 c shows the potential energy curve of the ^3^LE (red), ^3^CT (blue) and ^1^CT (black) states of PTZ‐DBTO2 along a mass‐frequency‐scaled coordinate of its lowest frequency normal mode (*v*_1_=14 cm^−1^). This mode, found to be important for the ISC/rISC dynamics, corresponds to a twist of the donor–acceptor dihedral angle, as shown schematically in Figure 2 b. In Figure 2 c, the filled circles correspond to quantum chemistry calculations obtained using time‐dependent density functional theory (TDDFT) within the Tamm–Dancoff approximation^29^ and the M062X exchange and correlation functional.^30^ The lines are a fit of the linear vibronic coupling Hamiltonian^32^ used to determine the nonadiabatic coupling between the two triplet states. Importantly, this fit reveals significant coupling between the two triplet states along this mode. This is also found to be the case for two additional lower frequency normal modes (*v*_11_ and *v*_23_), which are shown in Figure S1.

The fitted potential energy curves shown in Figure 2 c and Figure S1 are used to construct a three‐mode–three‐state model Hamiltonian, which is detailed in full in Table S3 and adopted in the proceeding dynamics simulations. Using this model, Figure 3 a shows the relative population of the ^3^LE state for 1 ns after excitation of the ^1^CT state; therefore, these dynamics follow the intersystem crossing (ISC) kinetics, simulated using the standard wavefunction formalism of the multi‐configurational time‐dependent Hartree (MCTDH) approach. The black trace uses the model Hamiltonian as calculated for PTZ‐DBTO2 (Table S3). This shows, after 1 ns, a population of the ^3^LE state of ≈0.075, and consequently, an ISC rate constant of *k*_ISC_=5×10^5^ s^−1^. Importantly, if the vibronic coupling between the ^3^LE and ^3^CT states is removed from the Hamiltonian (blue trace), *k*_ISC_ is significantly reduced and within the timescale of the dynamics population transfer to the ^3^LE (T_1_) state is negligible. Removing the HFI, which couples the two CT states (Figure 3 a inset) has very little effect on the ISC kinetics. This indicates, contrary to the recent conclusions of Ogiwara et al.,^23^ that the HFI cannot be the dominant mechanism. In contrast, increasing the vibronic coupling (red trace) or decreasing the energy gap (cyan trace) gives rise. as one might expect, to significant increases in *k*_ISC_, highlighting their dominant roles in the process.

**Figure 3:**
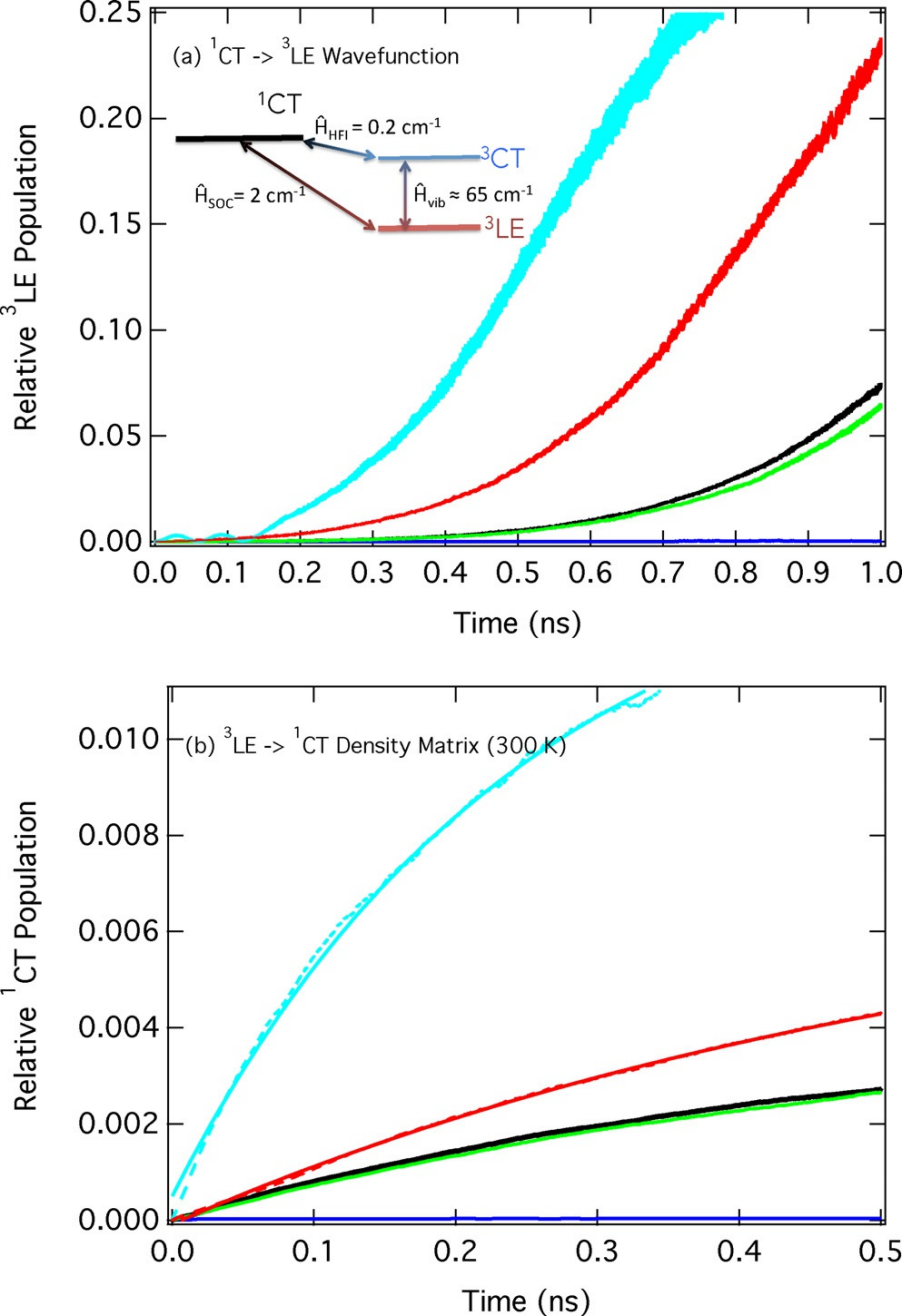
a) Relative populations of the ^3^LE state associated with intersystem crossing after excitation into the ^1^CT state. The inset is a simplified schematic representation of the model Hamiltonian used. b) The relative populations of the ^1^CT state associated with reverse intersystem crossing after initially populating the ^3^LE state. Black: full model Hamiltonian described in Table S3, green: no HFI, blue: No Vibronic Coupling, red: vibronic coupling increased by 10 %, cyan: energy gap between ^3^LE and ^3^CT halved.

Figure 3 b shows the same simulations, but in this case they are initiated in the lowest triplet state, ^3^LE, to mimic the rISC dynamics. Consequently, the population of the ^1^CT state is shown. These simulations are performed within the density operator formalism of MCTDH with a temperature of 300 K. As expected, these indicate the same trends as observed in Figure 3 a. The black trace finds a *k*_rISC_ 7×10^4^ s^−1^ and as expected, this can be correlated to *k*_ISC_ by $k_{\mathrm{rISC}}=k_{\mathrm{ISC}}exp\left(-\Delta E_{S_{1}-T_{1}}/k_{b}T\right)$ . If the vibronic coupling between the ^3^LE and ^3^CT states is again neglected (blue trace) the *k*_rISC_ is significantly reduced to a rate of ≈1×10^1^ s^−1^, in good agreement with recent calculations on similar systems by Chen et al.^26^ As seen from the schematic shown inset in Figure 3 a, in this case the *k*_rISC_ can only occur via the weak SOC between the ^3^LE and ^1^CT states, (2 cm^−1^). This population transfer can therefore be cast within Fermi's Golden rule, where the two excited states, ^3^LE and ^1^CT, are coupled via the SOC matrix element between them. Given the small magnitude of this coupling (2 cm^−1^) and the relatively large gap between the states (0.1 eV) it is not surprising that the rISC process in this case is slow.

Importantly, the critical role of vibronic coupling between the two triplet states for the ^3^LE→^1^CT conversion cannot be described within a similar first order perturbation theory approach. Therefore following the work of Henry and Siebrand,^33^ a more general expression of the ISC/rISC rate derived from second‐order perturbation theory must be adopted. Here the rISC rate is described as [Eq. (7)]:

(7) $$k_{i-f}^{\mathrm{rISC}}=\;\frac{2\pi }{\hbar }\sum_{f}{\left|\langle \Psi_{f}|{\hat{H}}_{\mathrm{int}}\left|\Psi_{i}\right\rangle +\sum_{n}\frac{\langle \Psi_{f}|{\hat{H}}_{\mathrm{int}}|\Psi_{n}\rangle \langle \Psi_{n}|{\hat{H}}_{\mathrm{int}}\left|\Psi_{i}\right\rangle }{E_{i}-E_{n}}\right|}^{2}\delta \left(E_{f}-E_{i}\right)$$

The first term is the normal first‐order Fermi′s Golden rule describing the transition from some initial state (Ψ_i_) to a final state (Ψ_f_). In contrast, coupling between the initial and final states in the second term, so called second order, is mediated by an intermediate state (Ψ_*n*_). In the present case, a direct second‐order coupling would require population transfer between the two CT states, via the HFI, that is, an initial ^3^LE state populates the ^3^CT via vibronic coupling, which decays into the ^1^CT, via the HFI. However, as already demonstrated, it plays an insignificant role. Consequently, the relevant terms for the present observations are [Eq. (8)]:

(8) $$k_{\mathrm{rIC}}=\frac{2\pi }{\hbar }{\left|\left\langle \Psi_{{}^{3}\mathrm{CT}}|{\hat{H}}_{\mathrm{vib}}|\Psi_{{}^{3}\mathrm{LE}}\right\rangle \right|}^{2}\delta \left(E_{{}^{3}\mathrm{CT}}-E_{{}^{3}\mathrm{LE}}\right)$$

and [Eq. (7)]:

(9) $$k_{\mathrm{rISC}}=\frac{2\pi }{\hbar }{\left|\frac{\langle \Psi_{{}^{1}\mathrm{CT}}|{\hat{H}}_{\mathrm{soc}}|\Psi_{{}^{3}\mathrm{LE}}\rangle \langle \Psi_{{}^{3}\mathrm{LE}}|{\hat{H}}_{\mathrm{vib}}\left|\Psi_{{}^{3}\mathrm{CT}}\right\rangle }{E_{{}^{3}\mathrm{CT}}-E_{{}^{3}\mathrm{LE}}}\right|}^{2}\delta \left(E_{{}^{1}\mathrm{CT}}-E_{{}^{3}\mathrm{LE}}\right)$$

Equations (6) and (7) indicate a two‐step mechanism. Firstly, the large vibronic coupling between ^3^LE and ^3^CT promotes, on a timescale much faster than the rISC (see Figure S2), an equilibrium between the two states. Obviously, the position of this equilibrium depends both on the size of the vibronic coupling and the energy gap. Subsequently, the second‐order term, Equation (7), couples the ^3^CT and the ^1^CT, using the ^3^LE as an intermediate. This latter second‐order term is very efficient because of the good vibrational overlap between the almost degenerate initial and final states, ^3^CT and ^1^CT, respectively. Therefore, the two coupling terms driving this dynamics are the SOC and the vibronic coupling elements. This explains recent experimental results which demonstrated that steric hindrance of D–A dihedral angle switches the main pathway from TADF to phosphorescent.^19^, ^21^, ^25^ This steric hindrance is equivalent to removing the vibronic coupling term, which is shown herein to be strongest along modes exhibiting a distortion of the D–A dihedral angle.

In the context of recent results on similar molecules with the D–A–D setup, Santos et al.^25^ reported a strong dependence on the relative energy levels according to the environment. This is not surprising, given the interplay between CT and LE states, both of which will exhibit different responses to changes in the local embedding environment. This gives rise to three distinct scenarios, shown schematically in Figure 4: a) the LE below the CT states, b) the LE degenerate with the CT states and c) the LE above the CT states. In the first case (Figure 4 a), the situation is as reported here. Assuming population exists only in lowest triplet state, a first step from ^3^LE to ^3^CT is required. This is then followed by a direct second order coupling via the initially populated ^3^LE state. For the latter two examples (Figure 4 b,c), a first step is not necessary and TADF can proceed efficient via a direct second‐order coupling mechanism. However, it is important to stress, as highlighted by the schematic in Figure 3 a and the population kinetics in Figure S2, that because the vibronic coupling is an order of magnitude larger than the other coupling mechanisms, this first step required in Figure 4 a will be significantly faster than the *k*_rISC_ process. It therefore is not the rate‐determining step and consequently will not change the overall rate of rISC. Moreover, rISC is independent of the location of the lowest energy ^3^LE state. For the present case, PTZ‐DBTO2, the ^3^LE is localised on the donor fragment. But recently an efficient TADF emitter has been confirmed with the lowest energy ^3^LE state residing on the acceptor fragment.^31^ Our present model, which can easily be recalculated to other D–A TADF molecular emitters, applies equally to this situation as the model only considers the lowest triplet excited state, not its location, and so explains rISC in the more general context of all D–A and D–A–D TADF emitters.

**Figure 4:**
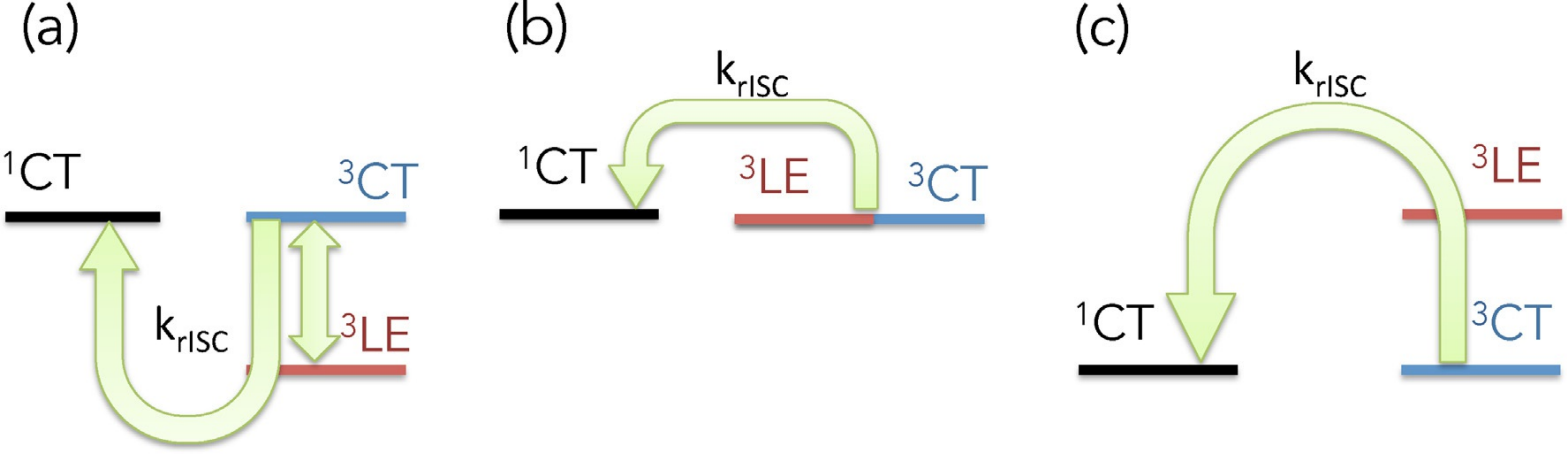
Schematic energy level diagrams illustrating the effect of the environment of the relative splitting of the CT and LE states of PTZ‐DBTO2.

In conclusion, our present results clearly demonstrate the critical contribution of non‐Born‐Oppenheimer effects to the rate of rISC in TADF molecules. Indeed, we show that a mechanism involving the conversion between two states is slow. Consequently, rISC is most effective between ^3^LE and ^1^CT states when vibronic coupling exists between the ^3^LE and the ^3^CT states. This gives rise to strong second order coupling. Crucially, these results, which explain recent experimental observations, reveal the dynamical mechanism of rISC and therefore great care must be taken in reducing non‐radiative decay by restricting molecular vibrations. This also demonstrates the important of not just tuning the Δ*E* ${}_{S{}_{1}\text{-}T{}_{1}}$ gap but playing close attention to the gaps between the ^3^LE and ^1^CT and ^3^CT and ^1^CT states. Finally, this emphasises the need for molecular design of novel functional materials that is based upon simulations that go beyond the Born‐Oppenheimer approximation and the limitations of the static picture provided by quantum chemistry.

## Computational Methods

The Hamiltonian used is based upon the vibronic coupling Hamiltonian^32^ and is described in detail in the Supporting Information. The potential energy surfaces were calculated using TDDFT(M062X)^30^ using a def2‐TZVP basis set^34^ as implemented within the Gaussian quantum chemistry package.^35^ The SOC matrix elements were computed using the perturbative approach^36^ implemented within ADF.^37^ The quantum dynamics were performed using the Heidelberg Multi Configuration Time Dependent Hartree (MCTDH) package.^28^ Further details are provided in the Supporting Information.

## Supporting information

As a service to our authors and readers, this journal provides supporting information supplied by the authors. Such materials are peer reviewed and may be re‐organized for online delivery, but are not copy‐edited or typeset. Technical support issues arising from supporting information (other than missing files) should be addressed to the authors.

Supplementary

## References

[1] Y. Wan , Z. Guo , T. Zhu , S. Yan , J. Johnson , L. Huang , Nat. Chem. 2015, 7, 785–792. 26391077 10.1038/nchem.2348

[2] W. Chang , D. N. Congreve , E. Hontz , M. E. Bahlke , D. P. McMahon , S. Reineke , T. C. Wu , V. Bulovic , T. Van Voorhis , M. A. Baldo , Nat. Commun. 2015, 6, 6415. 25762410 10.1038/ncomms7415

[3] A. Endo , M. Ogasawara , A. Takahashi , D. Yokoyama , Y. Kato , C. Adachi , Adv. Mater. 2009, 21, 4802–4806. 21049498 10.1002/adma.200900983

[4] M. A. Baldo , D. F. O'Brien , Y. You , A. Shoustikov , S. Sibley , M. E. Thompson , S. R. Forrest , Nature 1998, 395, 151–154.

[5] R. S. Nobuyasu, Z. Ren, G. C. Griffiths, A. S. Batsanov, P. Data, S. Yan, A. P. Monkman, M. R. Bryce, F. B. Dias, *Adv. Opt. Mater* **2016**, DOI: 10.1002/adom.201500689.

[6] M. N. Berberan-Santos , J. M. M. Garcia , J. Am. Chem. Soc. 1996, 118, 9391–9394.

[7] H. Uoyama , K. Goushi , K. Shizu , H. Nomura , C. Adachi , Nature 2012, 492, 234–238. 23235877 10.1038/nature11687

[8] C. Adachi , Jpn. J. Appl. Phys. 2014, 53, 060101.

[9] H. Beens , A. Weller , Acta Phys. Pol. 1968, 34, 593–542.

[10] Q. Zhang , H. Kuwabara , W. J. Potscavage, Jr. , S. Huang , Y. Hatae , T. Shibata , C. Adachi , J. Am. Chem. Soc. 2014, 136, 18070–18081. 25469624 10.1021/ja510144h

[11] T. J. Penfold , J. Phys. Chem. C 2015, 119, 13535–13544.

[12] S. Hirata , Y. Sakai , K. Masui , H. Tanaka , S. Y. Lee , H. Nomura , N. Nakamura , M. Yasumatsu , H. Nakanotani , Q. Zhang , K. Shizu , H. Miyazaki , C. Adachi , Nat. Mater. 2015, 14, 330–336. 25485987 10.1038/nmat4154

[13] D. R. Lee , B. S. Kim , C. W. Lee , Y. Im , K. S. Yook , S. H. Hwang , J. Y. Lee , ACS Appl. Mater. Interfaces 2015, 7, 9625–9629. 25924007 10.1021/acsami.5b01220

[14] X. Xiong , F. Song , J. Wang , Y. Zhang , Y. Xue , L. Sun , N. Jiang , P. Gao , L. Tian , X. Peng , J. Am. Chem. Soc. 2014, 136, 9590–9597. 24936960 10.1021/ja502292p

[15] C. A. Parker , C. G. Hatchard , Trans. Faraday Soc. 1961, 57, 1894–1904.

[16] C. Baleizão , M. N. Berberan-Santos , J. Chem. Phys. 2007, 126, 204510. 17552781 10.1063/1.2734974

[17] F. B. Dias , Philos. Trans. R. So., A 2015, 373, 20140447.

[18] M. Inoue , T. Serevičius , H. Nakanotani , K. Yoshida , T. Matsushima , S. Juršėnas , C. Adachi , Chem. Phys. Lett. 2016, 644, 62–67.

[19] J. S. Ward , R. S. Nobuyasu , A. S. Batsanov , P. Data , A. P. Monkman , F. B. Dias , M. R. Bryce , Chem. Commun. 2016, 52, 2612–2615. 10.1039/c5cc09645f 26750426

[20] B. T. Lim , S. Okajima , A. K. Chandra , E. C. Lim , Chem. Phys. Lett. 1981, 79, 22–27.

[21] F. B. Dias , K. B. Bourdakos , V. Jankus , K. C. Moss , K. T. Kamtekar , V. Bhalla , J. Santos , M. R. Bryce , A. P. Monkman , Adv. Mater. 2013, 25, 3707–3714. 23703877 10.1002/adma.201300753

[22] Q. Zhang , J. Li , K. Shizu , S. Huang , S. Hirata , H. Miyazaki , C. Adachi , J. Am. Chem. Soc. 2012, 134, 14706–14709. 22931361 10.1021/ja306538w

[23] T. Ogiwara , Y. Wakikawa , T. Ikoma , J. Phys. Chem. A 2015, 119, 3415–3418. 25774790 10.1021/acs.jpca.5b02253

[24] E. Hontz , W. Chang , D. N. Congreve , V. Bulović , M. A. Baldo , T. Van Voorhis , J. Phys. Chem. C 2015, 119, 25591–25597.

[25] P. L. Santos , J. S. Ward , P. Data , A. S. Batsanov , M. R. Bryce , F. B. Dias , A. P. Monkman , J. Mater. Chem. C 2016, 4, 3815–3824.

[26] X. K. Chen , S. F. Zhang , J. X. Fan , A. M. Ren , J. Phys. Chem. C 2015, 119, 9728–9733.

[27] C. M. Marian , J. Phys. Chem. C 2016, 120, 3715–3721.

[28] M. H. Beck , A. Jäckle , G. A. Worth , H.-D. Meyer , Phys. Rep. 2000, 324, 1–105.

[29] S. Hirata , M. Head-Gordon , Chem. Phys. Lett. 1999, 314, 291–299.

[30] Y. Zhao , D. G. Truhlar , Theor. Chem. Acc. 2008, 120, 215–241.

[31] P. Data, P. Pander, M. Okazaki, Y. Takeda, S. Minakata, A. P. Monkman, *Angew. Chem. Int. Ed* **2016**, DOI: 10.1002/anie.201600113. 10.1002/anie.201600113 27060474

[32] H. Köppel , W. Domcke , L. S. Cederbaum , Adv. Chem. Phys. 1984, 57, 59–246.

[33] B. R. Henry , W. Siebrand , J. Chem. Phys. 1971, 54, 1072.

[34] F. Weigend , R. Ahlrichs , Phys. Chem. Chem. Phys. 2005, 7, 3297–3305. 16240044 10.1039/b508541a

[35] M. J. Frisch, G. W. Trucks, H. B. Schlegel, G. E. Scuseria, M. A. Robb, J. R. Cheeseman, G. Scalmani, V. Barone, B. Mennucci, G. A. Petersson, H. Nakatsuji, M. Caricato, X. Li, H. P. Hratchian, A. F. Izmaylov, J. Bloino, G. Zheng, J. L. Sonnenberg, M. Hada, M. Ehara, K. Toyota, R. Fukuda, J. Hasegawa, M. Ishida, T. Nakajima, Y. Honda, O. Kitao, H. Nakai, T. Vreven, J. A. Montgomery, Jr., J. E. Peralta, F. Ogliaro, M. Bearpark, J. J. Heyd, E. Brothers, K. N. Kudin, V. N. Staroverov, R. Kobayashi, J. Normand, K. Raghavachari, A. Rendell, J. C. Burant, S. S. Iyengar, J. Tomasi, M. Cossi, N. Rega, J. M. Millam, M. Klene, J. E. Knox, J. B. Cross, V. Bakken, C. Adamo, J. Jaramillo, R. Gomperts, R. E. Stratmann, O. Yazyev, A. J. Austin, R. Cammi, C. Pomelli, J. W. Ochterski, R. L. Martin, K. Morokuma, V. G. Zakrzewski, G. A. Voth, P. Salvador, J. J. Dannenberg, S. Dapprich, A. D. Daniels, Ö. Farkas, J. B. Foresman, J. V. Ortiz, J. Cioslowski, D. J. Fox, Gaussian 09, Revision a 1, Gaussian Inc., Wallingford, CT, **2009**.

[36] F. Wang , T. Ziegler , J. Chem. Phys. 2005, 123, 154102. 16252937 10.1063/1.2061187

[37] ADF2009.01, SCM, Theoretical Chemistry, Vrije Universiteit, Amsterdam, The Netherlands. Scientifici Computation and Modelling, **2010**. http://www.scm.com/.

