# Supplementary material for: The Importance of Vibronic Coupling for Efficient Reverse Intersystem Crossing in Thermally Activated Delayed Fluorescence Molecules
Source: Chemphyschem. 2016 Jul 26;17(19):2956–61. doi: 10.1002/cphc.201600662 (PMC5096030; doi:10.1002/cphc.201600662)
Supplement: Supplementary file 1 — Supplementary [file CPHC-17-2956-s001.pdf]

CHEMPHYSCHEM

Supporting Information

The Importance of Vibronic Coupling for Efficient Reverse Intersystem Crossing in Thermally Activated Delayed Fluorescence Molecules

Jamie Gibson,[a] Andrew P. Monkman,[b] and Thomas J. Penfold*[a]

cphc_201600662_sm_miscellaneous_information.pdf

S1 Linear Spin Vibronic Model Hamiltonian 2

S2 Quantum Dynamics Simulations 3

S3 Supplementary Results 5

List of Figures

S1 Potential energy curves along ν_{11} (left$_{a}nd\nu_{23}$ (right) for the low lying excited states (^{3}LE = red, ^{3}CT = blue and ^{1}CT = black) relative to the ^{3}LE energy minimum and the corresponding fit of the model vibronic coupling Hamiltonian to these potentials. The nuclear motion corresponding to these modes are shown below. 5

S2 (a) Population kinetics of the ^{3}CT state during the rISC dynamics after initial population of the ^{3}LE state. These simulations are performed within the density operator formalisms with a temperature of 300 K and correspond to the same simulations as shown in Figure 3b in the main text. (b) Zoom in to the first picosecond of (a). 6

List of Tables

S1 Mode symmetry and vibration energies (in cm^{-1}) for the normal modes of PTZ-DBTO2 found to promote significant vibronic coupling and which are included in the model Hamiltonian. 2

S2 The lowest excited states of PTZ-DBTO2 at the Franck-Condon geometry included in the model Hamiltonian 3

S3 Parameters for each MCTDH simulation. The simulation colours in the top row correspond to the simulations plotted in Figure 3 in the main text. All energies are in cm^{-1} unless stated otherwise. In green are highlighted the parameters which are adjusted during each simulation 3

S4 Computational details for the MCTDH simulations within both the wavefunction and density operator formalisms. N$_i$ is the number of primitive harmonic oscillator discrete variable representation (DVR) basis functions used to describe each mode. n$_i$ are the number of single-particle functions used to describe the wavepacket on each state. 4

S1 Linear Spin Vibronic Model Hamiltonian

The Hamiltonian used in the present work is based upon a Linear model Vibronic Coupling Hamiltonian described in ref. 1. Here the diabatic potentials, most appropriate for quantum dynamics simulations, are obtained by fixing a point at which the adiabatic and diabatic potentials are equivalent (i.e. the coupling is zero). This is chosen to be the Franck-Condon point. The Hamiltonian is then expanded up to first order as a Taylor series around this point $\mathbf{Q}_0$, using dimensionless (mass-frequency scaled) normal mode coordinates:

$$\mathbf{H} = \mathbf{H}^{(0)} + \mathbf{W}^{(0)} + \mathbf{W}^{(1)} \tag{S1}$$

The zeroth order term is the ground state harmonic oscillator approximation. The zeroth order coupling matrix contains the adiabatic state energies for each state at $\mathbf{Q}_0$, i.e. The Franck-Condon excitation energies. The elements of the first order linear coupling matrix ($\mathbf{W}^{(1)}$) are written:

$$W_{ij}^{(1)} = \sum_{\alpha} \langle \Phi_i(\mathbf{Q}_0) | \frac{\partial H_{el}}{\partial Q_\alpha} | \Phi_j(\mathbf{Q}_0) \rangle Q_\alpha \tag{S2}$$

In the present model only the off-diagonal elements ($\langle \Phi_i(\mathbf{Q}_0) | \frac{\partial H_{el}}{\partial Q_\alpha} | \Phi_j(\mathbf{Q}_0) \rangle$), usually represented by the symbol λ, are present and act between the two triplet states (^{3}CT and ^{3}LE).

An important consideration when setting up a model Hamiltonian for large polyatomic molecules is which nuclear degrees of freedom are required in the model to ensure an accurate description of the dynamics of interest. Here we used symmetry considerations to determine all of the mode for which coupling between the two triplet states could be non-zero. At the equilibrium geometry PTZ-DBTO2 has C_s symmetry, and therefore the symmetry rule can be written:

$$\Gamma_i \otimes \Gamma_j \otimes \Gamma_\alpha \supset A' \tag{S3}$$

where Γ_i and Γ_j are symmetries of the two states and Γ_α is the symmetry of the mode. Therefore off diagonal elements (nonadiabatic coupling elements) will only be non-zero if the product of the two states gives the symmetry of the specific mode.

As the ^{3}CT and ^{3}LE states are of A" and A' symmetry, respectively only modes of A" symmetry will couple them. Subsequently all A" modes with a frequency $<$500 cm^{-1}, i.e. those which can reasonably be thermally activated, (15 in total) were calculated and fitted to determine the magnitude of non-adiabatic coupling. Only modes with significant coupling, > 50cm^{-1} were included. These are given in Table S1. The potentials are shown in Figure 2 in the main text and Figure S1.

Mode	Symmetry	Frequency (cm^{-1})	Character
1	A"	13.60	D-A bond rock
11	A"	173.65	A- Torsion
23	A"	422.60	D-A Torsion

Table S1: Mode symmetry and vibration energies (in cm^{-1}) for the normal modes of PTZ-DBTO2 found to promote significant vibronic coupling and which are included in the model Hamiltonian.

The potential energy surfaces were calculated using TDDFT(M062X) [2] within the Tamm-Dancoff approximation (TDA) [3] and a def2-TZVP basis set [4] as implemented within the Gaussian quantum chemistry package [5]. While TDDFT is well documented to have difficulties in addressing charge transfer excitations, the large fraction (54%) of non local exact exchange incorporated within the M062X functional remedies, to a large extent, this problem [6]. In addition, the TDA is critical for avoiding problems associated with the triplet instability [7]. The excited state energies for the relevant excited states at the Franck-Condon geometry are shown in Table S2 and are in good agreement with the onset of the experimental absorption spectra [8–10]. The SOC matrix element between the ^{1}CT and ^{3}LE states were computed at the ground state equilibrium geometry using the perturbative

approach developed by Wang and Ziegler [11] as implemented within ADF [12–14]. Using the M062X functional and a TZP basis set for all atoms. Scalar relativistic effects were included using the ZORA [15,16] approximation. The SOC matrix element between the two charge transfer states was zero at the Franck-Condon point. The Hyperfine interaction matrix element is known to be small and throughout this work a constant value of 0.2 cm^{-1}, in constant with typical experimentally recorded values is used [17].

State	Symmetry	Energy (eV)	Character
S_1	A”	3.55	HOMO→LUMO
T_1	A’	3.45	HOMO→LUMO+3
T_2	A”	3.53	HOMO→LUMO

Table S2: The lowest excited states of PTZ-DBTO2 at the Franck-Condon geometry included in the model Hamiltonian

During the previous paragraphs, the calculations required to obtained the Linear Spin Vibronic Model Hamiltonian are given. In table S3, the full details of the Hamiltonian for which simulation in the main text is given. This has the general form:

$$\hat{H} = \begin{pmatrix} \frac{\omega}{2}\left(\frac{\partial^2}{\partial Q_i^2} + Q_i^2\right) + E^{rel}_{^3LE} & \lambda_{Q_i} & E_{SOC} \\ \lambda_{Q_i} & \frac{\omega}{2}\left(\frac{\partial^2}{\partial Q_i^2} + Q_i^2\right) + E^{rel}_{^3CT} & E_{HFI} \\ E_{SOC} & E_{HFI} & \frac{\omega}{2}\left(\frac{\partial^2}{\partial Q_i^2} + Q_i^2\right) + E^{rel}_{^1CT} \end{pmatrix} \quad \text{(S4)}$$

where Q_i is the nuclear degree of freedom and E^{rel} is the excited state energy, relative to the ^3LE state. This means that $E^{rel}_{^3LE}$ will be 0, while for $E^{rel}_{^3CT}$ and $E^{rel}_{^1CT}$, the energy represents the gap between this state and the ^3LE state.

Simulation	black	green	cyan	blue	red
ω_1	13.60	13.60	13.60	13.60	13.60
ω_{11}	173.65	173.65	173.65	173.65	173.65
ω_{23}	422.60	422.60	422.60	422.60	422.60
$E^{rel}_{^3LE}$ (eV)	0.00	0.00	0.00	0.00	0.00
$E^{rel}_{^3CT}$ (eV)	0.07	0.07	0.035	0.07	0.07
$E^{rel}_{^1CT}$ (eV)	0.10	0.10	0.05	0.10	0.10
E_{SOC}	2.00	2.00	2.00	2.00	2.00
E_{HFI}	0.20	0.00	0.20	0.20	0.20
λ_1	67.02	67.02	67.02	0.0000	73.73
λ_{11}	91.71	91.71	91.71	0.0000	100.90
λ_{23}	79.04	79.04	79.04	0.0000	86.95

Table S3: Parameters for each MCTDH simulation. The simulation colours in the top row correspond to the simulations plotted in Figure 3 in the main text. All energies are in cm^{-1} unless stated otherwise. In green are highlighted the parameters which are adjusted during each simulation

S2 Quantum Dynamics Simulations

The quantum dynamics addressing the intersystem crossing rate were performed using the Heidelberg Multi Configuration Time Dependent Hartree (MCTDH) package [18,19]. In this approach the wavefunction *ansatz* is

written as a linear combination of Hartree products:

$$\Psi(Q_1, ..., Q_f, t) = \sum_{j_1=1}^{n_1} ... \sum_{j_f=1}^{n_f} A_{j_1...j_f}(t) \prod_{k=1}^{f} \varphi_{j_k}^{(k)}(Q_k, t) \tag{S5}$$

where Q_1,...,Q_f are the nuclear coordinates, $A_{j_1...j_f}(t)$ are the time-dependent expansion coefficients and $\varphi_{j_k}^{(k)}$ are the time dependent basis functions for each k (degree of freedom), known as single particle functions (SPFs). The SPFs used in MCTDH have two advantages: (1) fewer are required as they are variationally determined (2) the functions can be multi-dimensional particles containing more than one degree of freedom thus reducing the effective number of degrees of freedom. The wavepacket simulation describes the evolution of a certain, well defined, initial state. However for a system at finite temperature, important in the context of the simulations addressing the rISC rate, there obviously exists a mixture of different thermally excited states.

To address this we perform simulations within a density operator formalism of MCTDH [20]. Here the single particle functions are replaced with single-particle density operators. Here we adopt a closed quantum system, this is to say that no dissipative operators are included and only the core Hamiltonian described above is used. In this representation the Liouville-von Neumann equation for the system is expressed:

$$\dot{\rho}(t) = -\frac{i}{\hbar}[H, \rho(t)] \tag{S6}$$

For these simulations, the advantage of MCTDH comes into its own. Although the model Hamiltonian used herein is relatively small, for the density operator simulations the dimensionality of the system formally doubles [21] significantly increasing the numerical treatment of the simulations.

The full details of both sets of MCTDH simulations are given in Table S4 and ensure convergence for the population kinetics for the entire simulations

Table S4: Computational details for the MCTDH simulations within both the wavefunction and density operator formalisms. N_i is the number of primitive harmonic oscillator discrete variable representation (DVR) basis functions used to describe each mode. n_i are the number of single-particle functions used to describe the wavepacket on each state.

	Modes	N_i	n_{S_1},n_{T_1},n_{T_2}
Wavefunction	ν_1	25	12,12,12
	ν_{11}	25	12,12,12
	ν_{23}	25	12,12,12
Density Operator	ν_1,ν_{11}	21	21,61,31
	ν_{23}	21	12,12,12

The dynamics were performed using the model Hamiltonian described above. The computational details for the quantum dynamics simulations are shown in Table S1 and ensured convergence of the population kinetics shown in the main text.

S3 Supplementary Results

xww

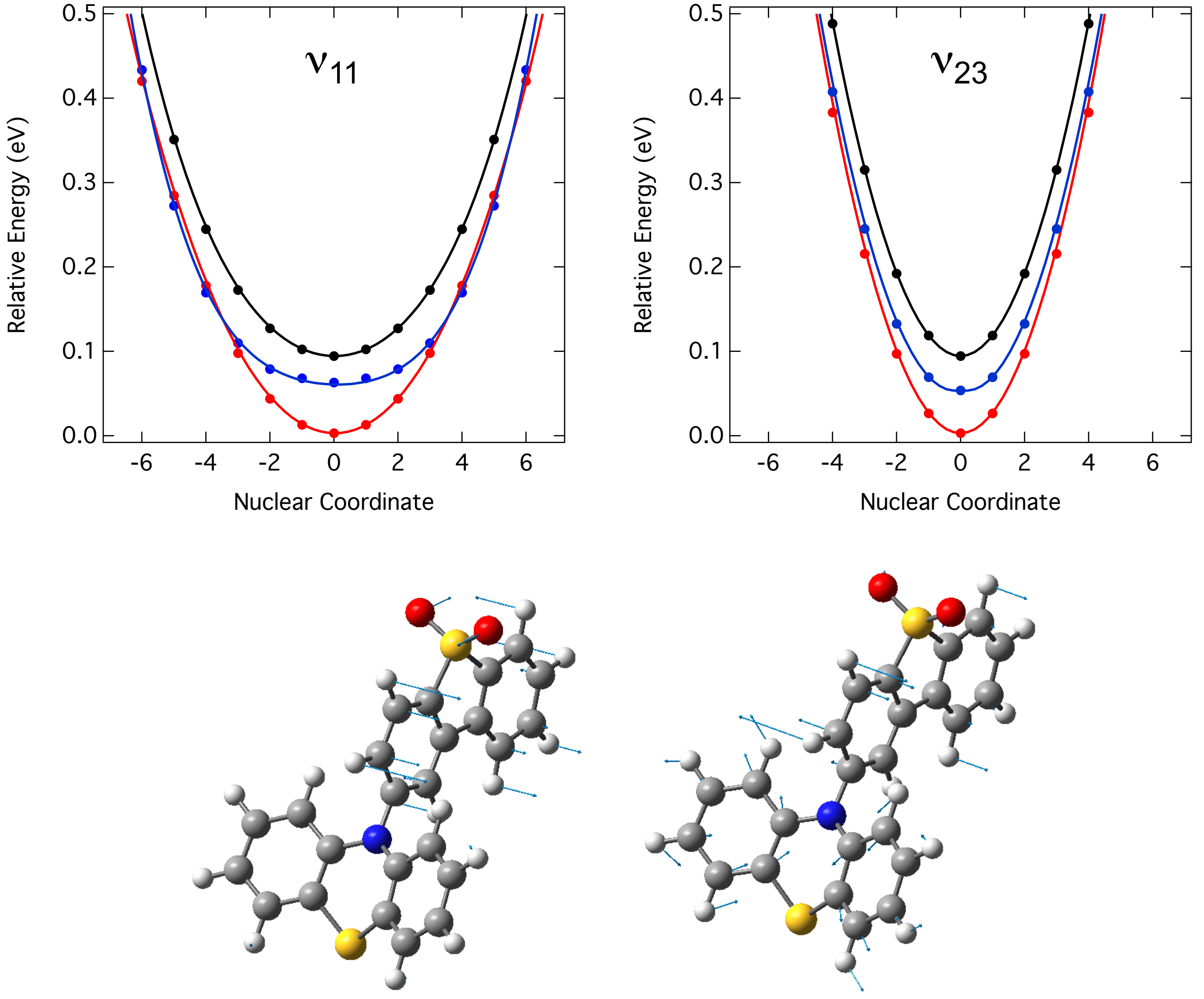

Figure S1: Potential energy curves along ν_{11} (left$_{a}nd\nu_{23}$ (right) for the low lying excited states (^{3}LE = red, ^{3}CT = blue and ^{1}CT = black) relative to the ^{3}LE energy minimum and the corresponding fit of the model vibronic coupling Hamiltonian to these potentials. The nuclear motion corresponding to these modes are shown below.

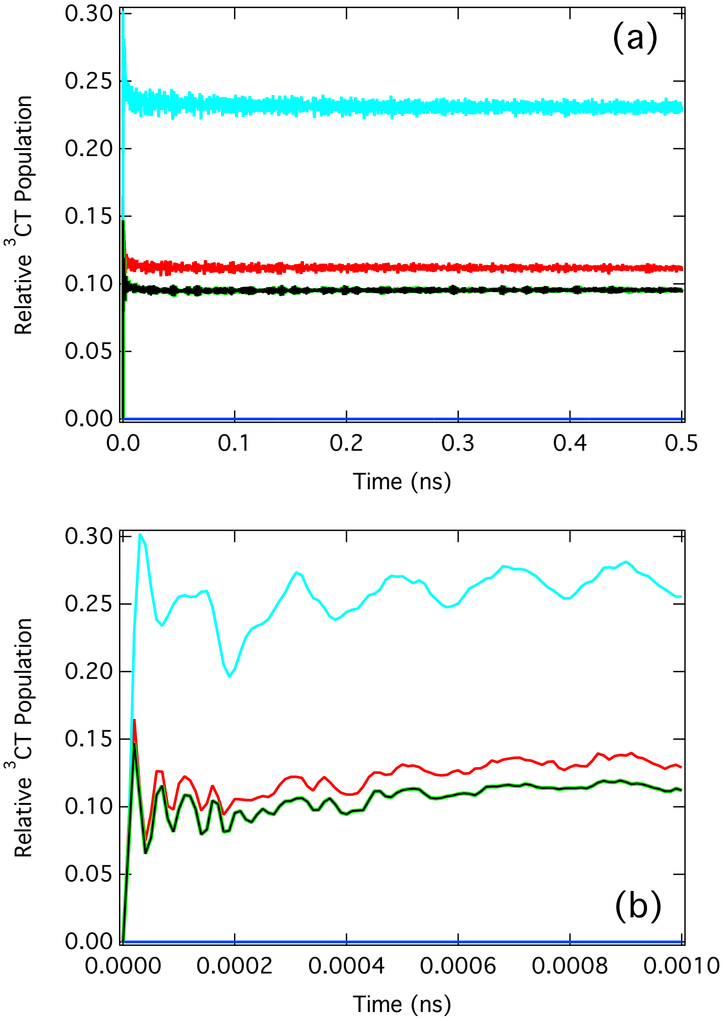

Figure S2: (a) Population kinetics of the ^{3}CT state during the rISC dynamics after initial population of the ^{3}LE state. These simulations are performed within the density operator formalisms with a temperature of 300 K and correspond to the same simulations as shown in Figure 3b in the main text. (b) Zoom in to the first picosecond of (a).

References

[1] H. Köppel, W. Domcke, and L. S. Cederbaum. *Adv. Chem. Phys.* **1984** 57:59–246.

[2] Y Zhao and D G Truhlar. *Theor. Chem. Acc.* **2008** 120:215–241.

[3] S. Hirata and M Head-Gordon. *Chem. Phys. Lett.*, **1999** 314:291–299.

[4] F Weigend and R Ahlrichs. *Phys. Chem. Chem. Phys.* **2005** 7:3297–3305.

[5] M. J. Frisch, G. W. Trucks, H. B. Schlegel, G. E. Scuseria, M. A. Robb, J. R. Cheeseman, G. Scalmani, V. Barone, B. Mennucci, G. A. Petersson, H. Nakatsuji, M. Caricato, X. Li, H. P. Hratchian, A. F. Izmaylov, J. Bloino, G. Zheng, J. L. Sonnenberg, M. Hada, M. Ehara, K. Toyota, R. Fukuda, J. Hasegawa, M. Ishida, T. Nakajima, Y. Honda, O. Kitao, H. Nakai, T. Vreven, J. A. Montgomery, Jr., J. E. Peralta, F. Ogliaro, M. Bearpark, J. J. Heyd, E. Brothers, K. N. Kudin, V. N. Staroverov, R. Kobayashi, J. Normand, K. Raghavachari, A. Rendell, J. C. Burant, S. S. Iyengar, J. Tomasi, M. Cossi, N. Rega, J. M. Millam, M. Klene, J. E. Knox, J. B. Cross, V. Bakken, C. Adamo, J. Jaramillo, R. Gomperts, R. E. Stratmann, O. Yazyev, A. J. Austin, R. Cammi, C. Pomelli, J. W. Ochterski, R. L. Martin, K. Morokuma, V. G. Zakrzewski, G. A. Voth, P. Salvador, J. J. Dannenberg, S. Dapprich, A. D. Daniels, Ö. Farkas, J. B. Foresman, J. V. Ortiz, J. Cioslowski, and D. J. Fox. Gaussian 09 revision a.1. Gaussian Inc. Wallingford CT 2009.

[6] R Li, J Zheng, and D G Truhlar. *Phys. Chem. Chem. Phys.* **2010** 12:12697–12701.

[7] MJG Peach, MJ Williamson, and DJ Tozer. *J. Chem. Theory Comput.* **2011** 7:3578–3585.

[8] JS Ward, RS Nobuyasu, AS Batsanov, P Data, AP Monkman, FB Dias, and MR Bryce. *Chem. Commun.*, **2016** 52:2612-2615.

[9] FB Dias, KN Bourdakos, V Jankus, K C Moss, K T Kamtekar, V Bhalla, J Santos, M R Bryce, and A P Monkman. *Adv. Mater.*, **2013** 25:3707–3714.

[10] PL Santos, JS Ward, P Data, AS Batsanov, MR Bryce, FB Dias, and AP Monkman *J. Mater. Chem. C*, **2016**, 4:3815-3824

[11] F Wang and T Ziegler. *J. Chem. Phys.* **2005** 123:154102.

[12] C Fonseca Guerra, J G Snijders, G Te Velde, and E J Baerends. *Theor. Chem. Acc.*, **1998** 99:391–403.

[13] S.J.A. van Gisbergen, J.G. Snijders, and E.J. Baerends. *Comput. Phys. Commun.*, **1999** 118:119–138.

[14] *ADF2009.01, SCM, Theoretical Chemistry, Vrije Universiteit, Amsterdam, The Netherlands*. Scientifici Computation and Modelling, 2010. http://www.scm.com/.

[15] E. van Lenthe, E. J. Baerends, and J. G. Snijders. *J. Chem. Phys.*, **1993** 99:4597–4610.

[16] E. van Lenthe, E. J. Baerends, and J. G. Snijders. *J. Chem. Phys.*, **1994** 101:9783–9792.

[17] T Ogiwara, Y Wakikawa, and T Ikoma. *J. Phys. Chem. A*, **2015** 119:3415–3418.

[18] H.-D. Meyer, U. Manthe, and L. S. Cederbaum. *Chem. Phys. Lett.* **1990** 165:73–78.

[19] M. H. Beck, A. Jäckle, G. A. Worth, and H.-D. Meyer. *Phys. Rep.*, **2000** 324:1–105.

[20] H-D Meyer and GA Worth. *Theor. Chem. Acc.*, **2003** 109:251–267.

[21] Michael Berman, Ronnie Kosloff, and Hillel Tal-Ezer. *J. Phys. A*, **1992** 25:1283.
